# Variation and disparity within the inner ear and trigeminus of the tenrecomorpha

**DOI:** 10.1038/s42003-025-08489-8

**Published:** 2025-07-23

**Authors:** R. Benjamin Sulser, Ross D. E. MacPhee

**Affiliations:** 1 Division of Evolutionary Ecology, Institute of Ecology and Evolution, Universität Bern, Bern, Switzerland; 2 Department of Mammalogy, American Museum of Natural History, New York, NY USA

**Keywords:** Evolution, Ecology, Zoology

## Abstract

Evolutionary theory predicts that sensory systems should adaptively respond to environmental selection. Different ecological niches should, in theory, then correlate with changes in sensory anatomy in lineages that have undergone extensive radiation. The afrotherian clade Tenrecomorpha, comprising of African potamogalines and Malagasy tenrecines, is of particular interest because of its variety: the clade reportedly includes fossorial, arboreal, semiaquatic, and even echolocating taxa. To investigate their sensory ecology, we provide geometric morphometric analyses of inner ear endocasts of 24 tenrec species. We expand this dataset with 9 iodine-stained specimens to study trigeminal organization. Although tenrecomorphs display cross-taxon differences in sensory structures, our analyses distinguish signals of conflicting strength and direction within the tenrec ear, with no single factor that might explain a substantial portion of observed variation when accounting for phylogeny. This contrasts with prior studies of the tenrec cranial endocast, where sensory ecotype and habitat are strongly associated with shape. Iodine-enhanced scans of the trigeminal nerve align with this, and other studies based on bony anatomy. The disparate patterns of shape evolution in Tenrecomorpha and the contrasts exhibited by the inner ear and trigeminal nerve provide a nuanced portrait of neurosensory adaptation, differing from expectations set by other mammalian groups.

## Abstract (web summary)

The inner ear and trigeminal nerves of tenrecomorph mammals are analyzed and compared; while sensory specialists may exhibit extreme features, the semicircular canals, cochlea, and trigeminal nerve do not share convergent evolutionary trajectories.

## Introduction

Interplay between phenotype and environment is an assumed feature of adaptive radiations, where ecological and morphological divergence are often correlated^1^. This assumption is logical but requires suitable testing. The afrotherian mammalian clade Tenrecomorpha is of particular interest for understanding the adaptive role of derived sensory behaviors, because its members display strong levels of functional convergence with other mammalian groups^2,3^. Consisting of only ~35 extant species^4^, tenrecomorphs embrace a wide range of disparate and distinct ecomorphotypes^5,6^, with fossorial, aquatic, arboreal, and perhaps even echolocating ecotypes^7,8^ and a range of locomotor behaviors^5^. Prior work focusing on osteology suggests that tenrecomorphs display surprisingly low cranial^9^ and mixed post-cranial^10–13^ intra-clade variation. By contrast, their cranial endocasts – which provide a proxy for brain shape and size – reflect strong signals of ecology and habitat that might be expected within such an adaptive radiation^14^ together with evidence of convergence with certain sensory specialists^2^.

In small mammals, the evolution of derived sensory behaviors are linked to significant adaptive modifications within bony and soft tissue organ structures, such as cochlear coiling in the ear^15^ or development of specialized vibrissae^3^. Recent advances in high-resolution x-ray computed microtomography (μCT) and geometric morphometric (GM) techniques have encouraged the development of multiple methods to quantify shape in these organs^16,17^. The bony labyrinth of the mammalian inner ear, consisting of the cochlea, vestibule, and semicircular canals, provide the essential anatomical substrate for hearing and balance.

Variations in labyrinth shape and size have functional significance, and are often utilized to identify ecological signals in both extant^2,18–21^ and extinct^22^ mammalian taxa. The vestibulocochlear nerve (cranial nerve VIII) splits into cochlear (spiral) and vestibular ganglia, extensions of which track changes in membranes via the sensitive hair cells lining the membranous labyrinth^23,24^. In terms of system function, it is thought that energy waves are refracted towards the cochlear apex. As the basilar membrane widens towards the tip of the cochlea, tighter coiling and an extended “apex” of the cochlea correlate with a propensity for low-frequency hearing^25^. Conversely, a less-coiled and comparatively thicker basal cochlear turn is often correlated with high-frequency hearing^26^. The three semicircular canals (lateral = LSC, anterior = ASC, and posterior = PSC) function in tandem with the otolith organs to determine balance, acceleration, and head position; relative changes in these canals are thought to be associated with changes in sensitivity and agility^27–30^. Increased degrees of curvature and increased radii within these canals correlate with an increase in afferent sensitivity to acceleration in the plane of the canal^31^, although the comparative strength of signal in these structures may be less than in other aspects of inner ear anatomy^32^ (and may be absent in some clades^33,34^). Of additional interest in regard to sensory ecology is the trigeminal nerve, which contains cell bodies of afferent nerves associated with sensory vibrissae^35^. Both systems have been implicated in adaptations to novel sensory environments^3,36^. The trigeminal nerve (cranial nerve V) runs from the trigeminal ganglion (consisting of both a motor and a sensory root), and splits into three main branches: the ophthalmic, maxillary, and mandibular. The maxillary branch directly innervates the mystacial vibrissae, key somatosensory structures in many mammals^15,37^.

The clade Tenrecomorpha encompasses ~35 species, usually framed taxonomically as a single family (Tenrecidae) with two constituent subfamilies, Potamogalinae and Tenrecinae^4,8^. These represent the extant continental African and Malagasy radiations of these taxa, respectively. Tenrecinae is further dived into three subclades (tribes): the “soft” tenrecs or Oryzorictini, the spiny tenrecs or Tenrecini, and their sister group, the monotypic Geogalini. Of the extant diversity, specimens of 24 species were available for hard-tissue aspects of this study, while another 9 were utilized for iodine-stained reconstructions. Sources for different ecologies and habitats can be found in previous literature^4,7,38^. It is worth emphasizing that tenrecomorph body masses range over several orders of magnitude, from *Microgale parvula* (3.3 g^39^) to *Tenrec ecaudatus* (887.59 g^39^, possibly up to 2 kg^40^).

This paper leverages an abundance of new data and methods to investigate shape variation within the inner ear and trigeminal nerve in tenrecomorphs, to the best of our knowledge many of which are presented here for the first time. Our sample includes a notable range of ecomorphotypes, from prehensile-tailed *Microgale longicaudata* to fossorial *Oryzorictes* sp. to semiaquatic *Microgale mergulus*. We use geometric morphometrics, quantitative statistics, and qualitative investigations of bony and stained tissue to directly test if the shape variation found in neuroanatomical structures across tenrecid species cane be meaningfully associated with their sensory ecomorphology, as previously suggested by a cranial endocast study^14^. Our investigation reveals how the morphospaces of these structures correspond to the ecology, phylogeny, and sensory behavior of these animals, as well as how they compare and contrast with patterns identified in other mammalian groups.

## Results

### The inner ears of the tenrecomorpha

**Fig. 1:**
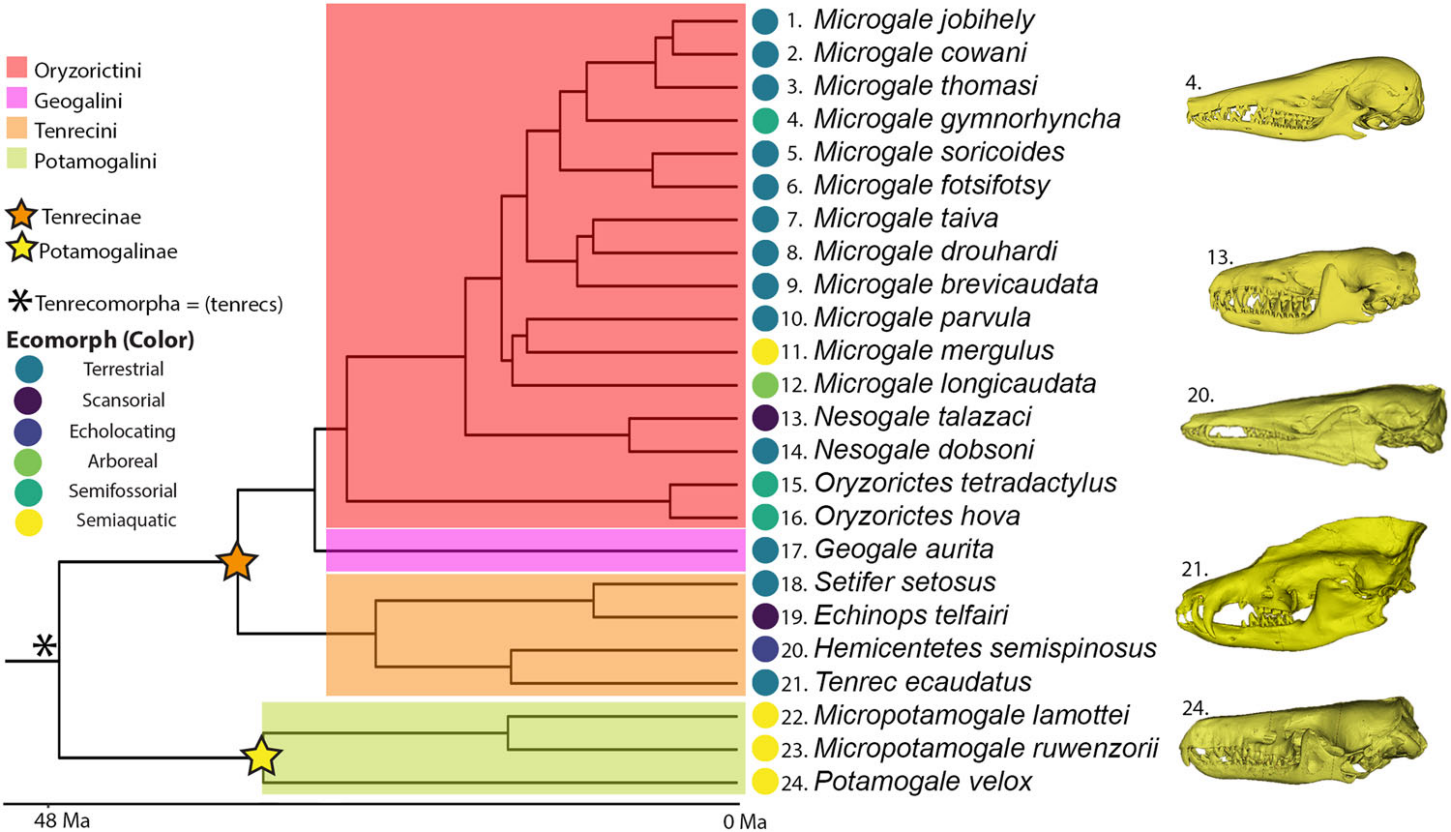
Phylogeny of tenrecomorph species under analysis. Illustrated phylogeny of the species included in this study. Branch lengths follow Everson et al. 2016, and designations follow Bronner et al. 2023. Clade Tenrecomorpha is designated by an asterisk (*). Tenrec subfamilies (Tenrecinae and Potamogalinae) are designated by orange and yellow stars, respectively. Tenrecine clades are designated by highlighted regions (Oryzorictini = red, Geogalini = pink, Tenrecini = orange, and Potamogalini = green).

**Fig. 2:**
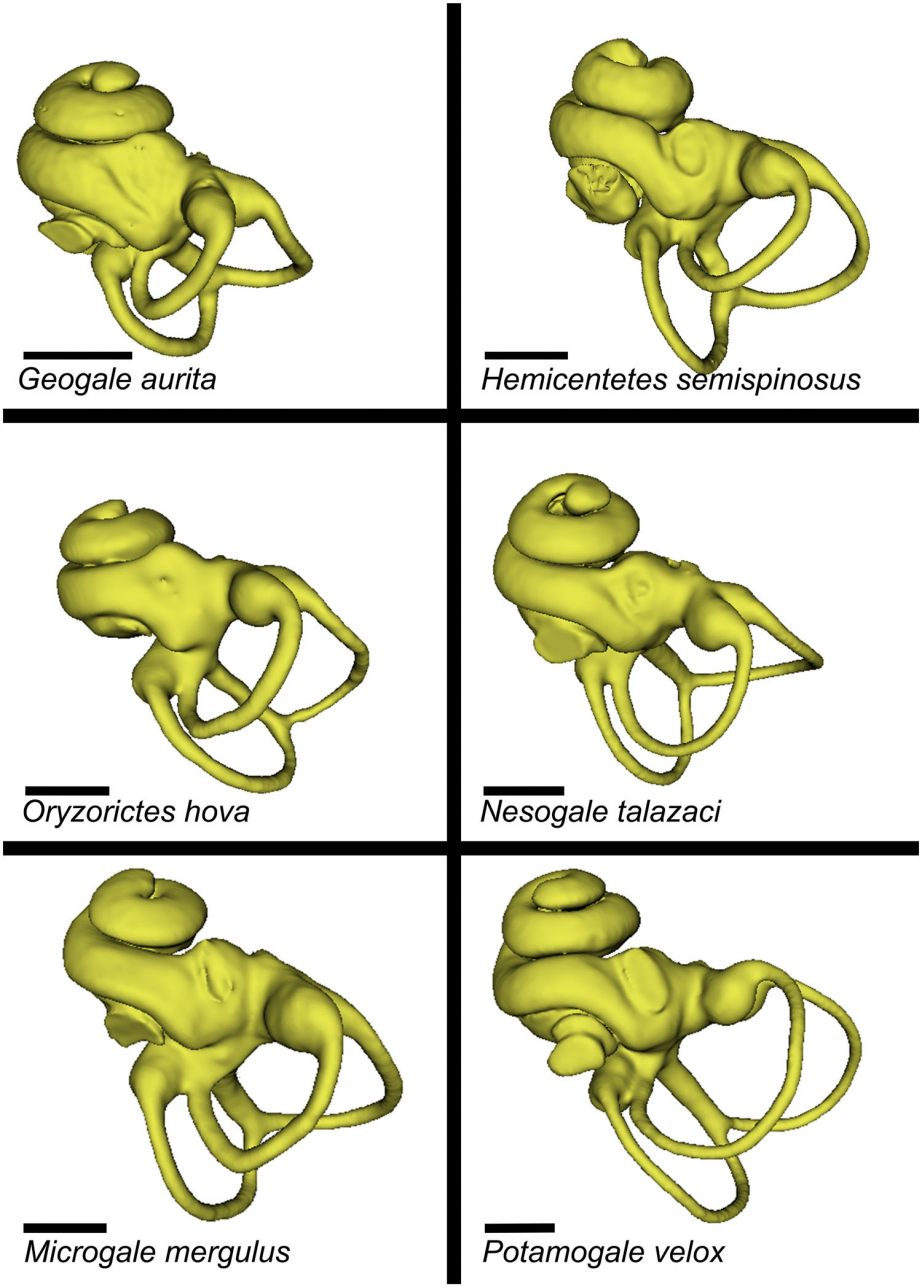
Illustration of inner ear bony labyrinth in selected taxa. Selected examples of tenrec inner ear endocasts created for this project, orientated to display all three semicircular canals and cochlear spiral. Additional meshes for all specimens used in this project can be found in Supplemental Data 1, with raw data available on MorphoSource. Scalebar = 1 mm.

Our sampling of tenrecid bony labyrinths reveals that the cranial disparity so evident within this clade is reflected in the inner ear. Broad-scale cladistic differences can be teased out from inner ear anatomy directly as well as our metrical analyses (For a list and phylogenetic positioning of each taxon included in this analysis, see Fig. 1, for images of selected species, see Fig. 2, for all models and shape data see Supplementary Data 1). Potomogalidae, as exemplified by *Potamogale velox*, show a ventrally flattened and tightly coiled cochlea associated with long, gracile semicircular canals. Shape differences are particularly noticeable with respect to the lateral canal, which is clearly inflected away from the cochlea in all specimens (and in the case of *Micropotamogale*, expanded in such a way that it makes contact with the margin of the posterior canal near the base of the ampulla – this “secondary common crus” has been observed rarely in placental mammals^41^). By contrast, the semicircular canals of fossorial Oryzorictini and *Geogale* are short, thick, and condensed, with a particularly small lateral canal and a broad basal cochlear turn (Fig. 2). In *Geogale* in particular, the canals are clearly reduced in relation to cochlear size, causing the bony labyrinth to have a curious, lopsided appearance. The cochlea appears enlarged and comparatively tightly coiled (although still less than the characteristic hyper-coiling observed in the related golden moles, Chrysochlorids^42^). Given these peculiar inner ear morphologies described in the Chrysochloridae, there is also interest in identifying if similar structures are present in the fossorial members of the tenrec clade. *Oryzorictes hova* and *O. tetradactylus*, the mole tenrecs, share a broad and relatively loosely coiled cochlea that sharply contrasts with the pattern found in their sister afrosoricid clades^42^. The semicircular canals are relatively short, with smaller radii in the anterior and posterior canals.

Within the tenrecini, the overarching semicircular canal patterns are similar, and the cochlea maintains a similar shape and coiling pattern for all taxa (although in *Hemicentetes semispinosus* the cochlea is slightly less tightly coiled). Finally, *Microgale* spp. display some notable disparities: *Microgale mergulus* is clearly distinct from potamogalids and congenerics alike, with a “loosely” coiled, small cochlea with shorter lateral canal radii than in any of its close relatives. *Microgale gymnorhyncha* is also known to share similar digging specializations^6^ with *Oryzorictes*, but does not appear especially distinct from other members of *Microgale* genus in terms of the bony labyrinth. In fact, the semicircular canals are quite broad, with the lateral canal nearly contacting the posterior, a trait shared by *M. longicaudata* and *M. thomasi* despite their different ecologies. After Procrustes alignment and ordination, all analyses of the dataset exhibited significant evolutionary signal (observed *K* = 0.700–0.717 and *P* value = 0.001, see Supplementary Fig. 1). The results of regression tests are provided in Supplementary Table 1. Regression analysis identified several factors (diet, centroid size, habitat, body mass, and ecomorphotype) as statistically significant under *P* < 0.01, with diet (including distinctions in specialized prey) as the best-fit factor across all partitions of the dataset (although only at *P* < 0.015 in the cochlea-only portion). When multiple factors are included in the model, the models that include centroid size + specialized diet and ecomorphology + habitat are the best fit in the full ear and semicircular datasets. The cochlea-only dataset only recovers a model encompassing centroid size and diet as statistically significant at *P* < 0.05 after accounting for multiple testing (see ANOVA results in Supplementary Table 1). Taking the shared phylogeny of the clade into account via PGLS drastically reduced the number of statistically significant factors across all partitions and analyses. The diet and habitat models are significant (in the full ear dataset, only habitat) at *P* < 0.05, and even this vanishes after adjustment for multiple testing (see Supplementary Table 1).

**Fig. 3:**
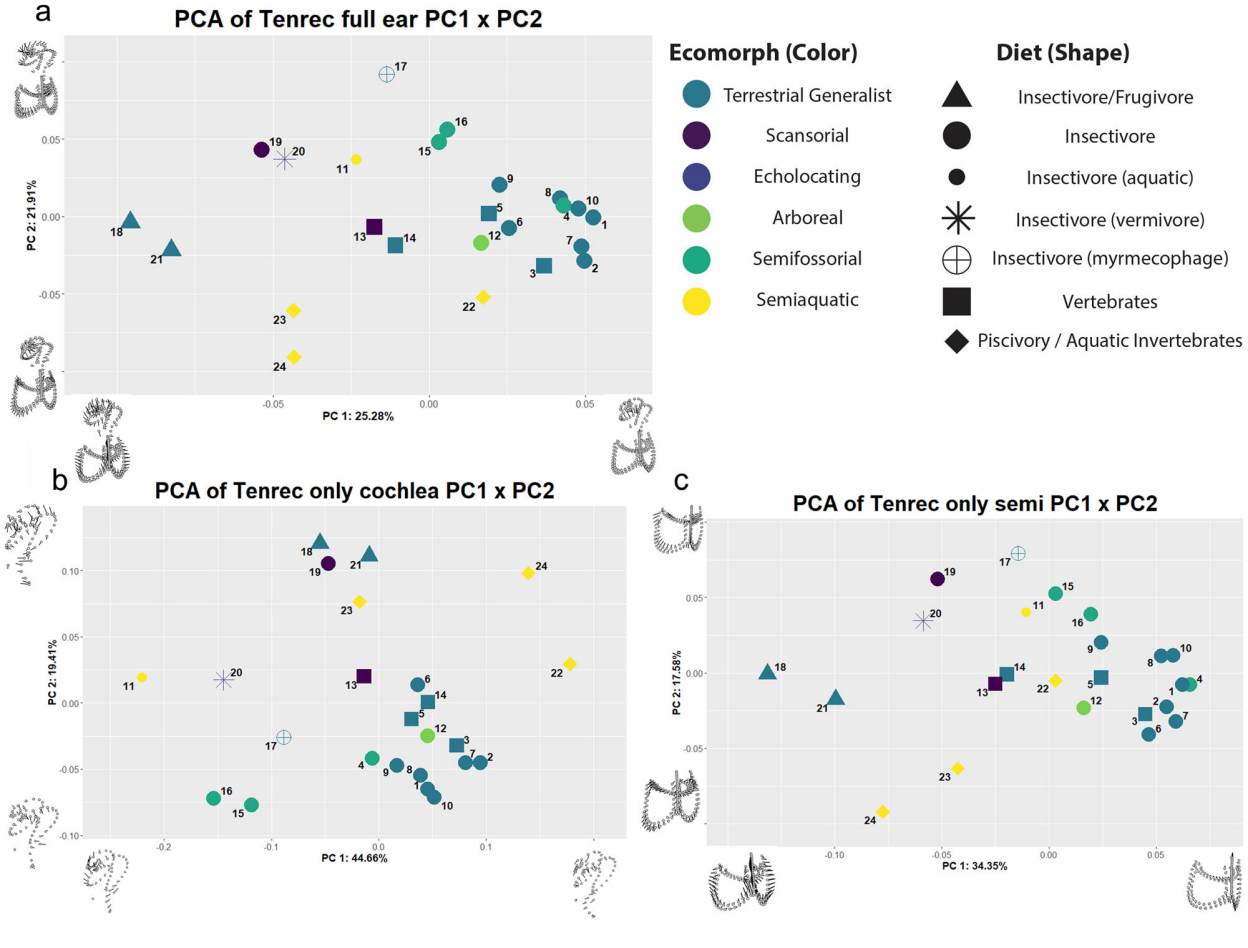
PCA results for the inner ear endocast. Morphospace results from the first two components of Principal Component Analyses on all inner ears used in this study. Full bony labyrinth (**a**), Cochlea-only (**b**), and Semicircular only (**c**) are visualized. All other components consisting of more than 10% of the total variation can be found in Supplementary Figs. 3–5. Taxa are characterized by color (indicating ecomorphotype; Blue = Terrestrial Generalist, Magenta = Scansorial, Purple = Echolocating, Light Green = Arboreal, Green = Semifossorial, Yellow = Semiaquatic) and shape (indicating diet; Triangle = Insectivore/Frugivore, Large Circle = Insectivore, Small Circle = Insectivore (aquatic insects), Asterisk = Insectivore (vermivore), Crossed Circle = Insectivore (myermecophage), Square = Vertebrates, Diamond = Piscivory/Aquatic Invertibrates). To aid in visualizing the representative shapes, sample vectors been created from the extreme of each positive and negative value as compared to the mean shape for each axes using the function plotRefToTarget in the Geomorph package (Adams and Otárola-Castillo, 2013) for each component and oriented for legibility for each axis of variation. Taxon and phylogenetic relationships for each data point can be found in Fig. 1.

For PCA groupings (Fig. 3), the bulk of the variation within the bony labyrinth lies in the first three components (PC1 = 25.28%, PC2 = 21.91%, PC3 = 12.72%, Fig. 3 and Supplementary Fig. 2)). All other components comprise less than 8% of the total variation. Minimum values of PC1 are associated with relatively narrower cochlear width, and broad canals in all directions without any twisting, while the maximum value relates with a broad cochlear width, and decidedly irregular canals (the lateral canal is notched inwards near the connection to the posterior canal, and the anterior canal is flared outwards). The minimum value of PC2 is associated with the Potamogalidae: very broad loops in all canals, with particularly wide expansion of the lateral canal, and tight cochlear coiling. The max PC2 value displays an especially enlarged cochlea with greatly reduced semicircular canals (exemplified by *Geogale*). Finally, the third principal component displays a larger loop in the anterior rather than the posterior canal, together with a much reduced cochlear coil in the minimum direction; the converse configuration (ASC similar to PSC; expanded cochlear size) applies to the maximum. Across the first two principal components, potamogalids express decreasing values along PC2, with *Oryzorictes* spp. and *Geogale* occupying particular regions of the morphospace. The cochlea-only dataset (Fig. 3b, Supplementary Note 1), on the other hand, suggests very different signals. PC1 refers largely to cochlear coiling and serves to distinguish the very different cochlear dimensions of the small, loosely coiled cochlea of *Microgale mergulus* from the tight coiling found in *M. ruwenzorii* and *Potamogale velox*. PC2 of the dataset is chiefly associated with cochlear width (similar to the cochlear signals found in PC1 in the full dataset). The semicircular canal-only data (Fig. 3c, Supplementary Note 1) shows similar results to the full ear dataset, segregating the large omnivorous tenrecs from the smaller insectivores along PC1, and contrasting the semiaquatic potamogalids from *Geogale* in PC2 (save for *Microgale lamottei*, which curiously groups closer to the center of the morphospace once the cochlea has been removed).

Due to the statistically significant influences of both allometry and phylogeny as determined via ANOVA and K-statistic tests, both signals were investigated further through adjustment. Allometry was minimized by developing an additional set of PCA plots on the regression of shape against log centroid size (Supplementary Fig. 2b, see Supplemental Note 2 for rationale). When accounting for allometry, the mainland African potamogalines are found tightly clustered within the broad *Microgale* envelope, while *Geogale aurita* groups closer to the tenrecini. It is of note that *Microgale mergulus* actually groups with species of *Oryzorictes* when this regression is performed. To better understand the impact of phylogeny, we ran phylogenetically aligned component analysis (PaCA) to visualize shape change concordant with phylogenetic signal (Supplementary Fig. 2c), which nearly matches the unadjusted PCA in terms of groupings, alongside other analyses to minimize phylogenetic effects (Phy-PCA, with adjusted residuals depicted in Supplementary Fig. 2d). Lending further support to the idea of a morphospace dominated by phylogenetic signal, when the effects of phylogeny are minimized much of the remaining signal evaporates, and most taxa cluster in the center of the dataset. By contrast, *M. mergulus* continues to separate from the rest of the dataset, a signal further enhanced once this analysis is adjusted to minimize phylogeny.

### The trigeminus of the tenrecomorpha

**Fig. 4:**
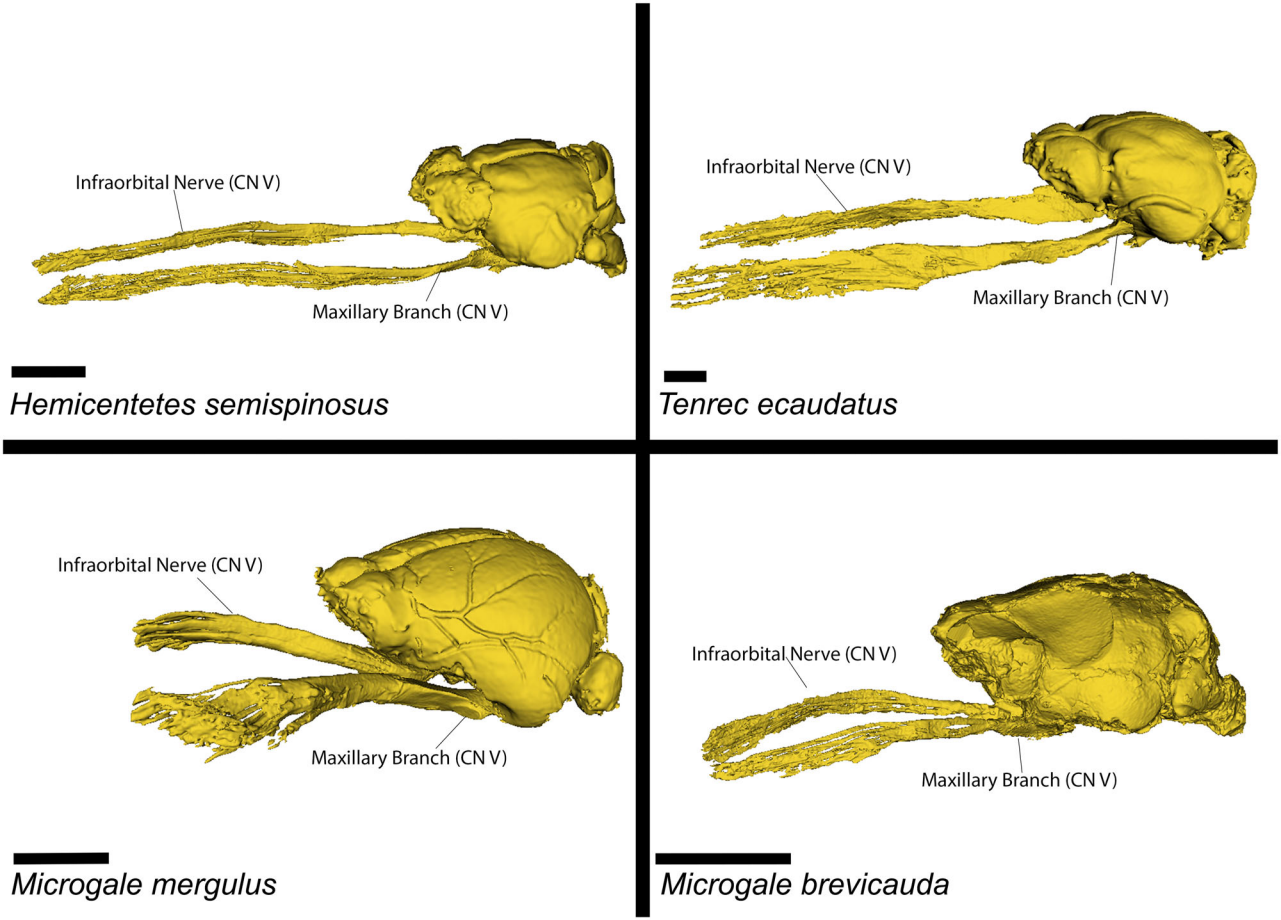
Iodine-stained Trigeminal Nerve in Selected Taxa. Reconstruction of the maxillary branch of the trigeminal nerve for selected examples of tenrec specimens, with the infraorbital nerve and maxillary branch of CN V indicated with a line. Raw data and additional species are available on MorphoSource. Scalebar = 5 mm.

**Fig. 5:**
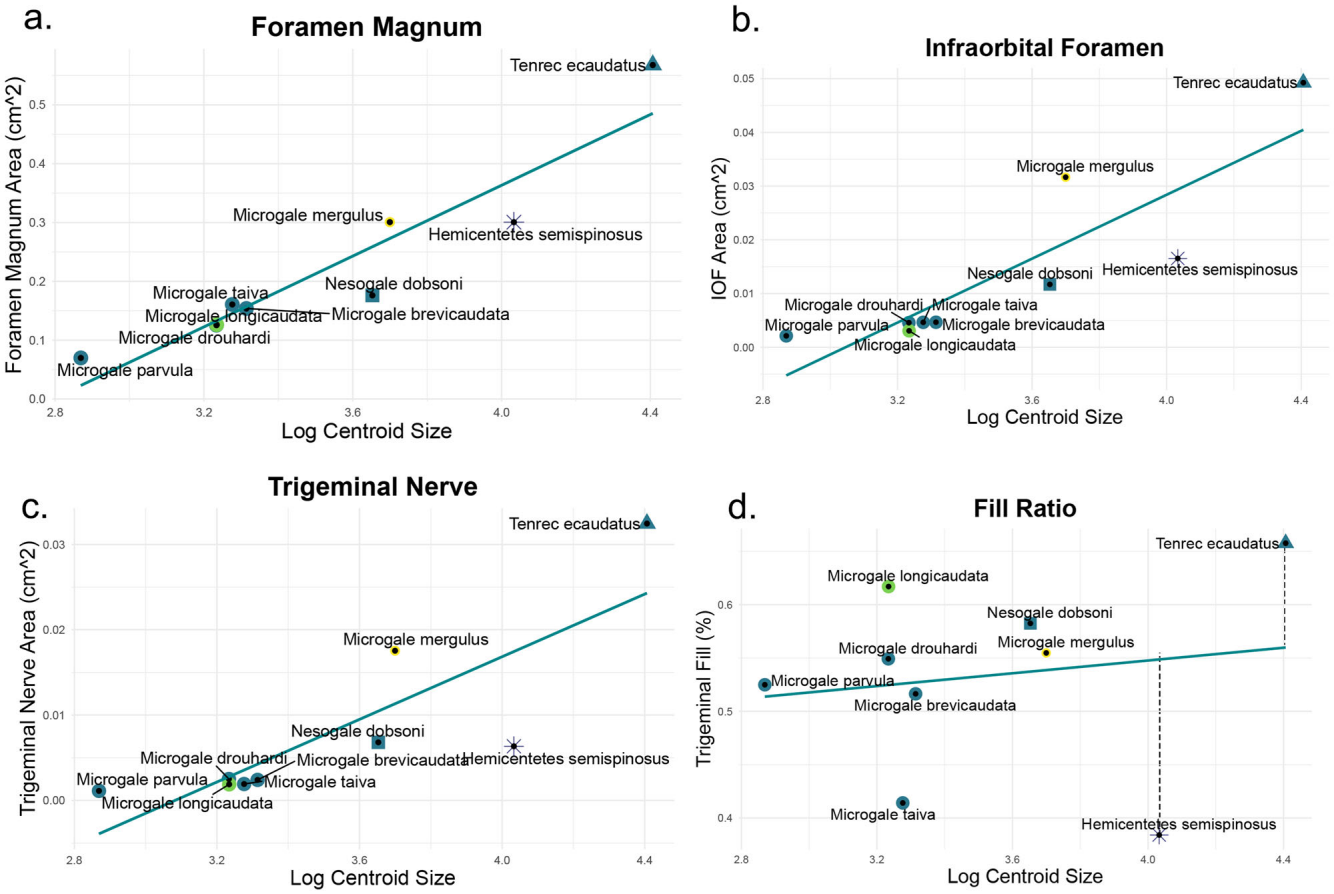
Linear regressions of bony and soft tissue indicia on centroid size. Linear regressions of various bony and soft tissue indicia on centroid size. Centroid size was calculated based on 9 landmarks around the skull (following Watanabe 2018) and plotted against the cross-sectional area of the foramen magnum (**a**), trigeminal nerve (**b**), and infraorbital foramen (**c**). All measurements were taken utilizing the Fiducials module in 3DSlicer. A fourth regression (**d**) was run to determine correlations between the infraorbital foramen cross-sectional area and that of the trigeminal nerve, with the largest and smallest values highlighted via a dashed line. Raw data and a list of taxa sampled are available in Supplementary Data 3.

Among the nine tenrecomorphs available for soft-tissue study, studies of the trigeminal nerve also provide contrasting signals between ecology and shape. The course of the trigeminal and maxillary nerve are depicted for selected species of interest in Fig. 4, rendered here along their origin until the nerves terminate into sensory vibrissae. Note that changes in curvature may largely result from differences in preparation and preservation, especially within the fleshy region of the snout at the anterior margin – this is perhaps most evident in *M. mergulus*, where much of the skin (including much of the vibrissae) was removed prior being made available for our study. The maxillary nerve follows a broadly similar course across all species scanned with this method, but relative size of the trigeminal in the semiaquatic *Microgale mergulus* is evident, as are the relative length of this nerve in the tenrecin specimens. Foramen magnum size correlates roughly with the body size of each specimen (as determined by the centroid^43^ (Fig. 5a); for identical analyses using published body mass, see Supplementary Fig. S5), while the infraorbital nerve through the infraorbital foramen show greater ecological differences relative to a size regression (Fig. 5b, c). As noted above, this nerve is not the only structures to pass through the infraorbital foramen, for this reason we also report the ratio of this space filled by the nervous tissue (hereafter, fill ratio). This fill ratio for all sampled tenrecomorphs is broadly similar and appears to hover around 40–60% (Fig. 5d), with the largest exceptions found in the tenrecids (see dotted lines). We interpret the space not inhabited by the nervous tissue most likely comes from vascular tissue, or space in the region ensuing from the shrinkage of these vascular elements due to water loss in the preservation and staining of these specimens. The conflicting signals between foramen magnum and the infraorbital foramen in this sample are in line with prior studies on these anatomical indices, which suggest independent size trajectories for each structure, and contrast with observations on similar small mammals^3^.

## Discussion

The sensory systems of Tenrecomorpha show several distinct (if not necessarily contrasting) signals which may be correlated with specific attributes. The semiaquatic behaviors of the potamogalines and *M. mergulus* are well documented in the literature^3,13,44^. Gracile semicircular canals, including a broad, ellipsoidal anterior canal and an inflected lateral canal, may be adaptations to a semiaquatic lifestyle, and may parallel changes observed in carnivorans and other mammalian taxa^2,19^. Yet despite obvious similarities in ecology and cranial endocasts, *Microgale mergulus* does not share the same cochlear patterning nor the same lateral canal sinuosity seen in potamogalines. Instead, this tenrec displays a much-shortened lateral canal and a comparatively squat and “loosely” coiled cochlear spiral. These differences in the *M. mergulus* inner ear cannot be explained by allometry alone (Supplementary Figs. S2bu–4b). It may be that this species acquired swimming behavior too recently for a strong signal to be reflected in otic morphology, or that significant behavioral differences exist between these two otherwise similar groups of tenrecomorphs. *M. mergulus* has been known to “sift” through the bottom of riverine habitats for food^44^, which suggests a fairly passive hunting strategy, whereas potamogalids are known to actively hunt fish and may therefore be more agile predators^45^. Such difference might place differing functional pressures on the inner ear.

Elsewhere in this morphospace, tenrecomorph inner ears deviate from expected patterns seen in other mammalian groups. *Hemicentetes semispinosus* and *Microgale longicaudata*, for instance, do not significantly vary from other tenrecines despite expressing proposed echolocation^46^ and specialized ambulatory behaviors^5,47^, respectively. Thus *H. semispinosus* might be expected to show (but does not) a large or expanded basal cochlear turn to maximize tuning in a high-frequency context. Similarly, the prehensile tail and marked climbing abilities of *M. longicaudata* might be expected to correlate (but does not) with an expanded radii looping semicircular canals relative to body size as seen in active climbing rodents and primates^27,48^.

Additionally, the comparison between the “full ear”, “cochlea only” and “semicircular canal” datasets do reveal important differences between these structures for their oft-utilized role in ecological inference. Clade clustering of the Potamogalinae, for instance, is only found in the complete ear analyses (Fig. 3a); in cochlear and semicircular canal-only datasets, these taxa pull away from each other in the morphospace (Fig. 3b, c). On the flipside, unique similarities in the *Oryzorictes* genus (and dissimilarities in *Microgale mergulus)* are clearly driven by their respective cochlear morphologies. While allometry is present across the entire ear, the stronger relationship to body mass and centroid size in the semicircular canal only dataset (albeit without accounting for phylogeny) is concordant with studies on other mammalian groups. The best fit models for OLS analyses are largely concordant with dietary preference across all three datasets, but the cochlear dataset does describe a decided lack of significant relationship for traits that might be expected to be reflected in the semicircular canals (locomotion and habitat).

Different parts of the ear may be associated with different aspects of dietary ecology, with the documented association of semicircular canals with movement and position (involved in prey capture) and cochlear spiraling related to sound processing (involved in prey identification or predator avoidance during foraging). The similarities of the “full ear” and the “semicircular canal” datasets and discrepancies with the “cochlear only” datasets suggest that, at least in this sample, aspects from one functional unit of the ear may swamp important signals in another. Previous studies of the cranial endocast^14^ suggested an overarching morphospace association with sensory ecology. This linkage was not detectable in this study of the bony labyrinth, indicating a disconnect between these two oft-studied sources of inference. The phylogenetic signals evident in the dataset are perhaps unsurprising given the relatively deep divergences amongst different clades^4^. However, the absence of significant convergent ecologically driven shifts in the semicircular canals and cochlea of the inner ear is notable, because the two semiaquatic ecomorphotypes in the clade represent long-separated lineages that nevertheless express convergence within cranial endocasts^14^ and foraminal attributes^3^. This lack of expected signal from the bony labyrinth may reflect the frequent weakness of signals recovered from this structure seen in some mammal clades^32,34^. Findings from the cochlear region are unexpected due to established links between cochlear coiling and hearing^21^. While some functional aspects of the inner ear may relate to specific diet-related shape patterns (for instance, it is possible the relatively large and tightly coiled cochlea of *Geogale* (Figs. 2, 3) may help the animal track the acoustic communication of termites^49^), the significant correlation observed for the “diet” category in linear models disappears if phylogeny is taken into account or if more generalized dietary categories are used (see Supplementary Table 1).

It may also be that shape changes identified in these structures are due to a constraint in skeletal and skull development rather than a functional directional signal, as has been observed in Chiroptera^50^. In any case, developmental and ethological studies will be needed to highlight the functional significance – if any – of such deviations in shape. These results should not be taken to mean that the morphospace signals related to diet or ecology are irrelevant for reconstructing the history of ecological adaptation in this clade. Rather, shape variation in the inner ear of tenrecomorphs is actually a patchwork of weak signals, roughly corresponding to the phylogenetic signal.

Study of the trigeminal nerves, using both bony correlates and the nerve itself, point to a similar conclusion. With respect to linear regressions of these metrics on centroid size (Fig. 5), oryzorictins largely show correlations in centroid size with cross-sectional area for all metrics (e.g., foramen magnum, infraorbital canal, and cross-sectional area of the trigeminal nerve), while the nerve is larger than expected for *Tenrec ecaudatus* and smaller than expected for *Hemicentetes semispinosus*. The larger and smaller fill ratios for these taxa also suggest that they respectively have a larger and smaller proportion of the infraorbital foramen filled by nervous tissue (these differences are also visible with respect to the nerve itself, see Fig. 4b). While broader sampling will be needed to investigate soft-tissue convergence across other tenrecomorph clades and species, these results suggest that ecologically specialized taxa may indeed exhibit some degree of disparity within both bone and soft tissue. For example, the aquatic *Microgale mergulus* and the “echolocating” *Hemicentetes semispinosus* have substantially larger and smaller trigeminal nerves and associated bony features, respectively, than would be expected for their body size. The genus *Oryzorictes* has a remarkably large foramen magnum relative even to the semiaquatic tenrecomorphs, but has been recorded as displaying a smaller brain relative to other taxa^51^. The trigeminal nerve in *Oryzorictes* has yet to be quantitatively investigated, but the neuroanatomical changes in these areas may be related to the fossorial behavior of this genus. Ecology is not always reflected in morphology, however. The arboreal *M. longicaudata* cannot be especially differentiated from its congenerics along any axis of cranial anatomy, although this taxa and the generalist *T. ecaudatus* appears to show an unexpectedly large cross-sectional area of the trigeminal nerve (a trait frequently observed in mammals with particular ecological specializations for somatosensation^3^). Likewise, the relatively unspecialized shrew tenrec *Microgale taiva* indicates a quite small fill ratio for its body size. Additional aspects of sensory anatomy such as visual systems and the brain regions directly responsible for the vestibular system may be of interest in a more targeted follow-up study.

Even considering the abundance of ecological specialists in the group, the tenrecomorph neurosensory system presents a complex mosaic of traits. While prior studies of this clade^9–13^ have investigated skeletal anatomy and the skull, the present findings directly investigate the bony and soft tissues of the cranium. First, the anatomy of these tenrecs does indeed vary in size and shape, from the squat and tightly coiled inner ear and semicircular canals of *Geogale aurita* to the elongated semicircular loops found in *Potamogale velox*. Extensions in potamogaline and reduction in geogalin semicircular canals set these tenrecomorphs apart and may relate to distinctive functional effects, such as increasing or reducing the afferent sensitivity of balance and movement to compensate for active swimming and slow movement, respectively. Second, in contrast to ecologically-driven shape variation found in the cranial endocast, geometric morphometric analyses show that when phylogeny is taken into account, size, diet, ecology, and habitat display a rather weak relationship with shape variation in the bony labyrinth across tenrecomorph species. This is also evident in the principal component morphospace, as the effects of semiaquatic adaptation clearly manifest differently in *Microgale mergulus* and *Potamogale velox*, particularly in the cochlea and the lateral semicircular canal. Additionally, any fossorial adaptations in *Oryzorictes* genera do not appear to be shared by the similarly fossorial *Microgale gymnorhyncha*. These findings do not change if the cochlea and semicircular canals are considered separately. Third, soft tissue and trigeminal nerve anatomy are remarkably consistent across the wide range of body sizes found in sampled oryzorictins, but there are sharp deviations in the sensory specialists and *Tenrec*. *Microgale mergulus* has large trigeminal structures, even if the inner ear differs from other semiaquatic tenrecomorph taxa. The proposed echolocating tenrec *Hemicentetes semispinosus* does not appear to display a bony labyrinth specialized for high-frequency hearing. The greatly reduced trigeminal neuroanatomical structures may, however, relate to the species’ derived stridulating behavior and sensory ecology if these reflect a compensatory shift away from somatosensation as a primary part of its sensory toolkit. Finally, the different neurosensory foci of the cochlea, semicircular canals, and trigeminus point to different patterns of adaptation within this clade. The data presented here suggest distinct selective pressures on distinct regions of the sensory apparatus in distinct groups, with unique specializations along each branch of the tenrec phylogeny rather than shared effects of a single overarching factor (such as sensory ecology) driving shape variation. These findings caution against making inferences about ecological adaptation from a single anatomical source. The variation of signals and structures within the Tenrecomorprha should inspire further investigation of anatomy and on long-isolated branches of the mammalian evolutionary tree.

## Methods

### Geometric morphometrics (GM)

**Fig. 6:**
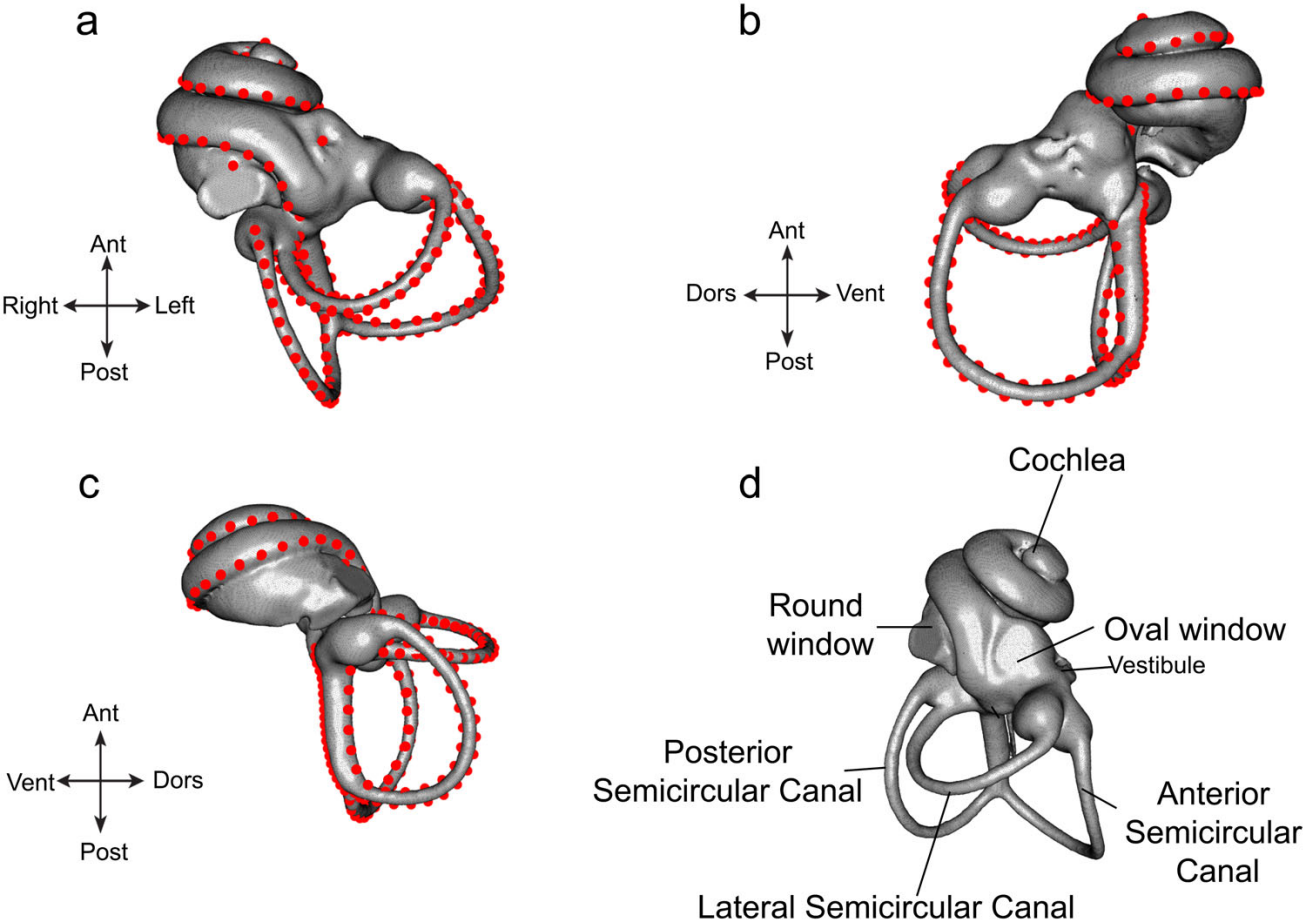
Illustration of landmarks for the inner ear bony labyrinth. Illustration of landmarks utilized for the inner ear bony labyrinth, represented on the left ear of *Nesogale talazaci* for **a** ventral, **b** medial, and **c** lateral views. For a detailed breakdown of landmark number and location, refer to Table 2. **d** Anatomical regions of interest within the inner ear cochlea.

Specimens for this project were provided from American Museum of Natural History (AMNH) and Field Museum of Natural History (FMNH), with permission from their respective collections departments. Skulls of 24 tenrecomorph species were scanned on the GE Phoenix Vtome x SMicroCT scanner in the AMNH Microscopy and Imaging Facility (MIF), at a target resolution between 18 and 56 μm (see Table 1 for a list of specimens). For all taxa, the left bony labyrinth of adult specimens was segmented. In the case of four specimens where this structure was damaged or otherwise unsuitable for analysis, the right cochlea was segmented and mirrored prior to analysis. For rendering, specimens were reconstructed with the 3DSlicer software suite^52^ and semilandmarks placed using the SlicerMorph package^53^. Landmarks placed in these analyses are listed in Table 2 and are shown in Fig. 6. For fixed landmarks, landmarks were collected^54^, translated from a template (*Nesogale talazaci*) using the ALPACA module^55^, and corrected/resampled by hand where necessary. Cochlear and semicircular landmarks were placed and resampled manually for each specimen, and all landmarks were merged to form the final dataset. Additionally, we compared the mirrored specimens with both the left and right ears of closely related species under this landmarking scheme to ensure that asymmetry within the left and right ears did not exceed the variance between species (see Supplementary Fig. 6).

**Table 1:** Species and specimens investigated in this study

Species name	Specimen ID	State
*Echinops telfairi*	AMNH 170599	Skull
*Geogale aurita*	FMNH 173143	Skull
*Hemicentetes semispinosus*	AMNH 275201	Skull, diceCT
*Microgale brevicaudata*	AMNH 275743	Skull
*Microgale brevicaudata*	AMNH 273705	diceCT
*Microgale cowani*	AMNH 275000	Skull
*Microgale drouhardi*	AMNH 275088	Skull
*Microgale drouhardi*	AMNH 275096	diceCT
*Microgale fotsifotsy*	AMNH 275281	Skull
*Microgale gymnorhyncha*	AMNH 275136	Skull
*Microgale jobihely*	AMNH 274987	Skull
*Microgale longicaudata*	AMNH 275145	Skull
*Microgale longicaudata*	AMNH 275209	diceCT
*Microgale mergulus*	AMNH 100689	Skull
*Microgale mergulus*	FMNH 165440	diceCT
*Microgale parvula*	AMNH 275364	Skull
*Microgale parvula*	AMNH 275154	diceCT
*Microgale soricoides*	AMNH 275297	Skull
*Microgale taiva*	AMNH 275298	Skull
*Microgale taiva*	AMNH 275174	diceCT
*Microgale thomasi*	AMNH 275357	Skull
*Micropotamogale lamottei*	FMNH 162893	Skull
*Micropotamogale ruwenzorii*	FMNH 207665	Skull
*Nesogale dobsoni*	AMNH 275209	Skull, diceCT
*Nesogale talazaci*	AMNH 207003	Skull
*Oryzorictes hova*	AMNH 275191	Skull
*Oryzorictes tetradactylus*	AMNH 31243	Skull
*Potamogale velox*	AMNH 5334	Skull
*Setifer setosus*	AMNH 170536	Skull
*Tenrec ecaudatus*	AMNH 170523	Skull, diceCT

**Table 2:** Landmarks utilized in this study

Point	Location	Type
1	Cochlear apex	Type II
2	Center of oval window	Type II
3	Center of round window	Type II
4	Bifurcation of lateral/anterior semicircular canals	Type I
5	Bifurcation of lateral/posterior semicircular canals	Type I
6	Bifurcation of posterior/anterior semicircular canals (common crus)	Type I
7–46	Cochlear spiral	Semilandmarks
47–66	Common crus	Semilandmarks
67–85, 66	Anterior canal	Semilandmarks
66, 86–104	Posterior canal	Semilandmarks
105–124	Lateral canal	Semilandmarks
7, 125–143	Internal Spiral (Margin of Scala Vestibuli)	Semilandmarks
144–163	Inside loop of Anterior canal and common crus	Semilandmarks
164–183	Inside loop of Posterior canal and common crus	Semilandmarks
184–203	Inside loop of Lateral canal	Semilandmarks

Geometric morphometric (GM) analyses were carried out in R^56^, with extensive use of the package *Geomorph*^57^. For all geometric morphometric data, coordinate points were aligned and scaled through Generalized Procrustes Analysis^58^ prior to any downstream analysis utilizing the function *gpagen*. To determine if different regions of the inner ear displayed different signals, as might be expected from the different functions of the inner ear, we also ran all analyses on cochlea-only and only semicircular-only landmark sets in addition to the full ear dataset.

### Statistics and reproducability

Potential explanatory factors were tested for statistical correlation with the resulting shape data. In addition to phylogenetic clades, shape changes within the bony labyrinth might be expected to correlate with locomotion strategy^19,27^, diet (particularly prey identification^59^ and hunting strategy^60^), and/or derived sensory behaviors such as echolocation^18^. A complete list of all factors analyzed, and citations for each, are presented in Supplementary Data 2. The number of unique instances and taxon sample sizes are highly variable between groups of traits. However, excessive lumping of dietary categories can obscure important ecological information^61^. To compensate for these concerns, both generalized and specific categories of diet were used in our analyses, with the more specific analysis differentiating (for example) aquatic prey items between fish and invertebrates at the cost of degrees of freedom in the model. Results should be interpreted with these considerations in mind.

Within the Geomorph package, OLS (Ordinary Least Squares), PGLS (Phylogenetically Generalized Least Squares), and ANOVA analyses were assessed to identify best-fit comparisons with the functions *ProcD.lm* and *ProcD.pgls*. All permutations of principle component analyses (PCA, phy-PCA, and PaCA) were performed utilizing *gm.prcomp*. For phylogenetic analyses, we used the topology of Everson et al.^4^ trimmed to the taxa analyzed in this study using the *ape*^62^ package in R, with the resulting variation tested for the presence evolutionary signal through computation of K-statistics^63^. Given the large size changes within the tenrec clade, shape coordinates with minimal allometric effects were visualized in two ways: by utilizing the residuals of the relationship between log centroid size and Procrustes-adjusted coordinates, and with analogous residuals between log body mass and coordinates (see Supplementary Table 1).

### Iodine staining

In addition to bony tissue, recent advances in the methodology of diffused iodine contrast-enhanced scanning (DiceCT) allow for the visualization and quantification of soft tissue structures with minimally invasive techniques^16,64–66^. These techniques are well suited to study of the trigeminal nerve, a structure essential for somatosensation and previously linked to a number of sensory specializations, particularly in aquatic and subterranean small mammals^3,51,67^. Infraorbital foramen size has been assumed to be a reasonable proxy for nerve size (and thus, acuity), and measuring it directly may help to validate this assumption. To measure the trigeminal nerve, nine species of adult tenrec were additionally scanned and reconstructed following iodine staining. Iodine staining followed the procedure of Gignac and Kley^64^; all specimens were scanned prior to staining, and then were submerged in 11% Lugol’s iodine solution for several days until the desired x-ray imaging contrast was achieved.

### Shrinkage and tissue quantification

While the potential shrinkage of neural tissues in the cranial region^68^ precludes using most morphometrics on regions of the brain itself (but see recent developments to mitigate this effect^66^), on historical specimens qualitative descriptions of the internal anatomy can be still be made with these techniques, corroborated where possible with published histological and anatomical records^51,69^. Structures with less available water in vivo (and thus, less availability for water loss post-treatment) such as bones^70,71^ or nerves^72^ should nonetheless still be applicable for use in morphometrics.

### Trigeminal nerve metrics

To quantify size differences in the trigeminal nerve and associated bony indices, a portion of the maxillary branch was segmented between the foramen rotundum and the cross-sectional position of the first molar. The trigeminal nerve was then aligned and measured using the function Segment Geometry in 3DSlicer to determine changes in nerve caliber and ensure that the cross sections measured were representative of this nerve as a whole in this region. Cross sectional area measurements of the infraorbital foramen, trigeminal nerve, and foramen magnum were then taken using the Fiducials module, as these areas have been shown to correlate with ecology and trigeminal size in other mammal groups^3^. Where applicable, measurements of paired structures (e.g., the left and right sides of the trigeminal nerve and infraorbital foramen) were averaged to obtain a single value. In addition to these area measurements, because these foramina also transmit vascular structures, a parametric fill ratio (trigeminal area/infraorbital area) was calculated to determine how trigeminal nerve size corresponds to its bony correlates (Supplementary Data 3). To investigate the effects of allometry, these nerve measurements and to ensure comparability to prior studies, nine landmarks were taken on the skulls of each stained specimen to estimate centroid size of the skull^43,73^. The resulting shape data are also included as a factor in linear regression analyses, in addition to body mass estimates taken from panTHERIA for each species^39^ (Supplementary Data 3).

### Reporting summary

Further information on research design is available in the Nature Portfolio Reporting Summary linked to this article.

## Supplementary information

Supplemental Material

Description of Additional Supplementary Files

Supplementary Data 1

Supplementary Data 2

Supplementary Data 3

Reporting Summary

## Data Availability

All data, including meshes and landmarks, that support this study are included in Supplementary Data 1 or on the associated Dryad repository^74^ (10.5061/dryad.4qrfj6qjn). Raw image data are available on MorphoSource (Project ID: 000706865).

## Code availability

Code to replicate all analyses in this manuscript are included alongside this study as supplemental files. All analyses were run under R^56^ 4.4.1. Code is also available on the Dryad repository^74^ (DOI: 10.5061/dryad.4qrfj6qjn).
