# Supplementary material for: Variation and disparity within the inner ear and trigeminus of the tenrecomorpha
Source: Commun Biol. 2025 Jul 23;8:1090. doi: 10.1038/s42003-025-08489-8 (PMC12284173; doi:10.1038/s42003-025-08489-8)
Supplement: Supplementary file 1 — Supplemental Material [file 42003_2025_8489_MOESM1_ESM.pdf]

Variation and Disparity within the Inner Ear and Trigeminus of the Tenrecomorpha

COMMSBIO- 24-3573

Supplementary Note

Supplemental Note 1: Additional notes on PC3 for partitioned analyses

PC3 of Semicircular Canals: Refers to the angle of the lateral canal and size of the anterior canal, with a larger ASC than PSC and inflected LSC in *Microgale mergulus* in the minimum and equivalent ASC and PSC (and level LSC) in the maximum, such as in *Oryzorictes* species. PC3 of Cochlea: Refers to the size and coiling of the apical turn, with a large cochlear volume of the apical turn in *M. ruwenzorii* and a much smaller cochlear turn in *H. semispinosus*.

Supplemental Note 2: Additional notes on allometric adjustment

Given the immense size range found within the clade, there is interest in determining the nature of the allometric signal in this dataset and how this may differ in different groups. Malagasy Tenrecines and Potamogalines nonetheless do not show a significant interaction between family and either metrics of size – centroid or mass – with the shape coordinates used in this study. As such, visualizing shape coordinates with minimal allometric effects were visualized under a common allometry.

A challenge with partitioning cladistic groups further within the tenrecomorpha is the monotypic genus *Geogale* – as the Geogalini consists of a single data point in our sample, we cannot create trendlines for tenrecs at or below the tribe level without excluding this taxon. A separate analysis excluding this species focusing on tribe that does suggest there may be a significant interaction at the tribe level at P >.05, but not at the family level (although we caution that this effectively a different dataset then the rest of the paper as a result of the species' removal). See Supplementary Table 1 for the reports from linear analyses.

A more targeted study of the variation in body size among this clade, with weights taken for individuals and multiple individuals for each taxon, would be intriguing but is beyond the bounds of what is possible from the data available for this study.

Supplementary Figures

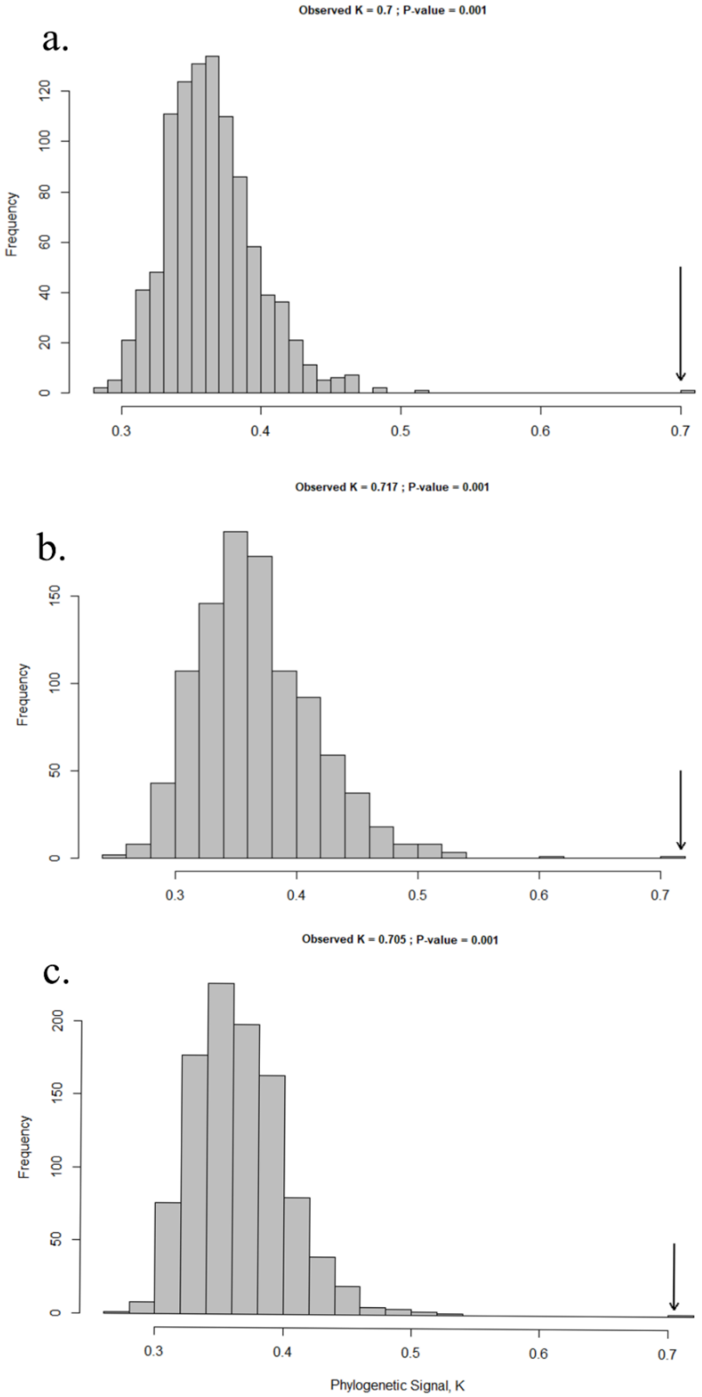

Supplementary Figure 1: Comparison of K values across the full ear (**a**), cochlea-only (**b**), and semicircular canal-only (**c**) datasets.

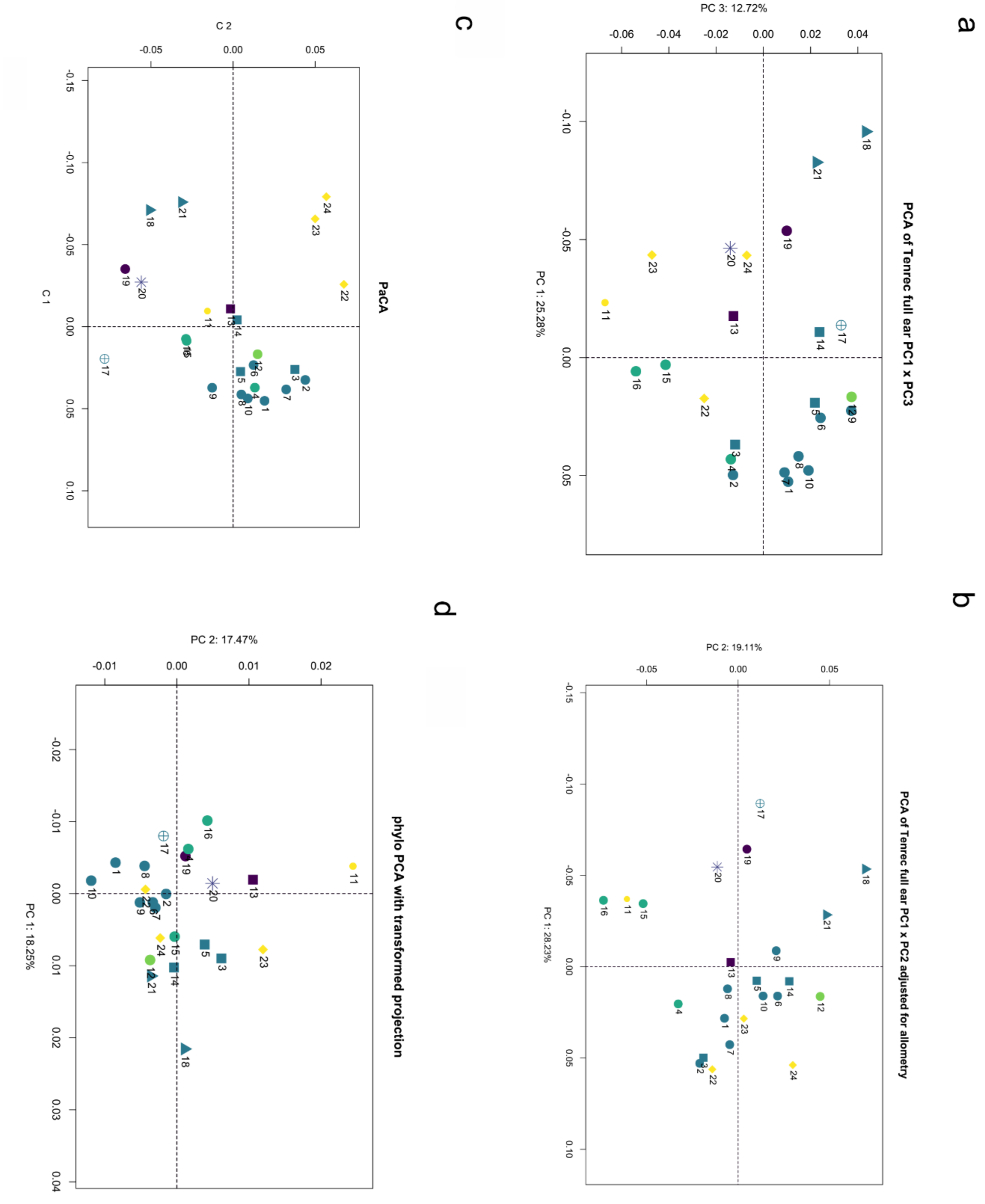

Supplementary Figure 2: Additional analyses of the full bony labyrinth for **a**. Standard PCA of the first and third principal component), **b**. Standard PCA under allometric adjustment under a common allometry, **c**. Phylogenetically aligned Component Analysis (PaCA), and **d.** Phylogenetic PCA (with transformed projection of residuals). Taxa are characterized by color (indicating ecomorphotype) and shape (indicating diet). Taxon and phylogenetic relationships for each data point can be found in Figure 1, key for shape and color can be found in Figure 3.

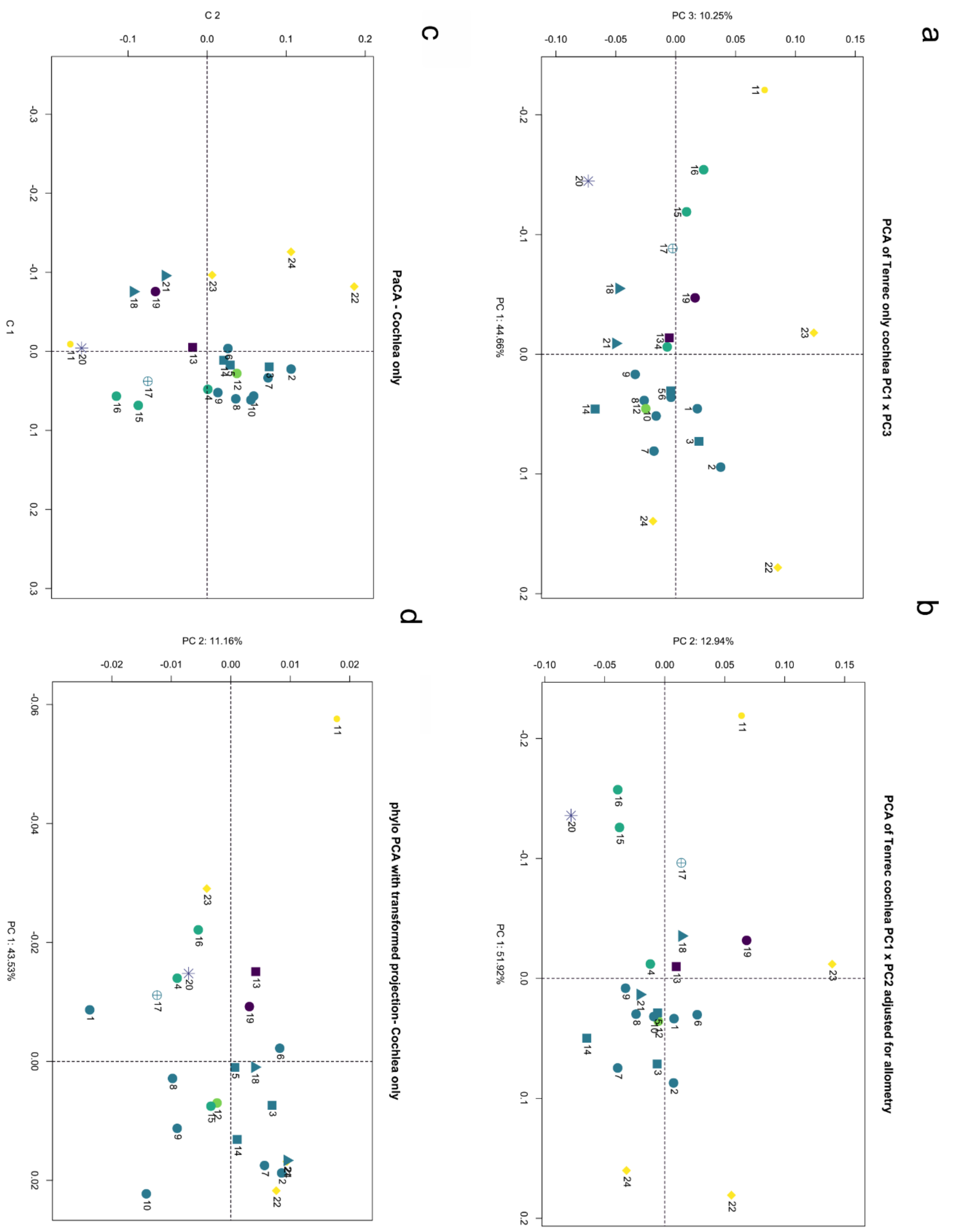

Supplementary Figure 3: Additional analyses of the cochlea only for **a**. Standard PCA of the first and third principal component), **b**. Standard PCA under allometric adjustment under a common allometry, **c**. Phylogenetically aligned Component Analysis (PaCA), and **d.** Phylogenetic PCA (with transformed projection of residuals). Taxa are characterized by color (indicating ecomorphotype) and shape (indicating diet). Taxon and phylogenetic relationships for each data point can be found in Figure 1, key for shape and color can be found in Figure 3.

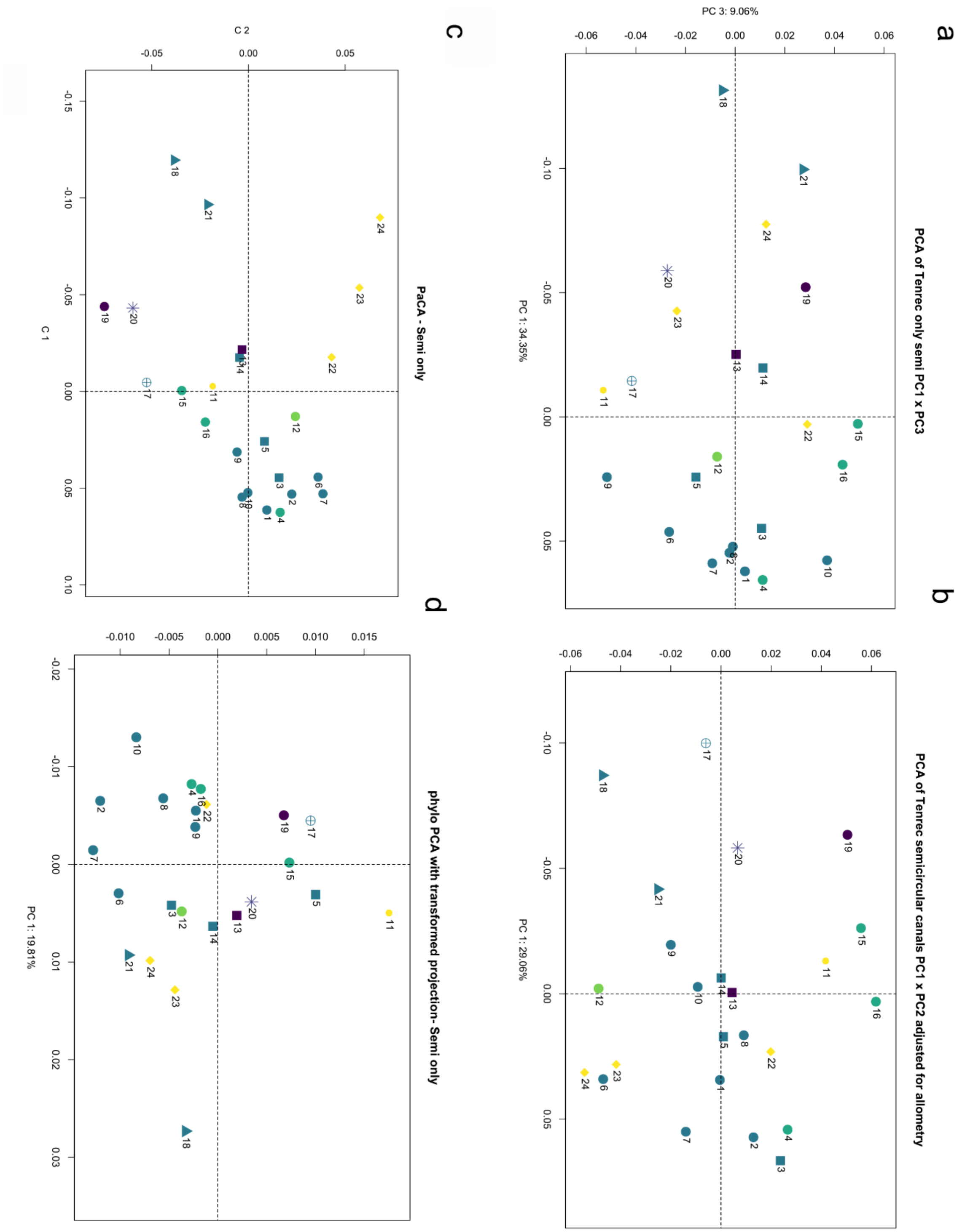

Supplementary Figure 4: Additional analyses of the semicircular canals only for **a**. Standard PCA of the first and third principal component), **b**. Standard PCA under allometric adjustment under a common allometry, **c**. Phylogenetically aligned Component Analysis (PaCA), and **d.** Phylogenetic PCA (with transformed projection of residuals). Taxa are characterized by color (indicating ecomorphotype) and shape (indicating diet). Taxon and phylogenetic relationships for each data point can be found in Figure 1, key for shape and color can be found in Figure 3.

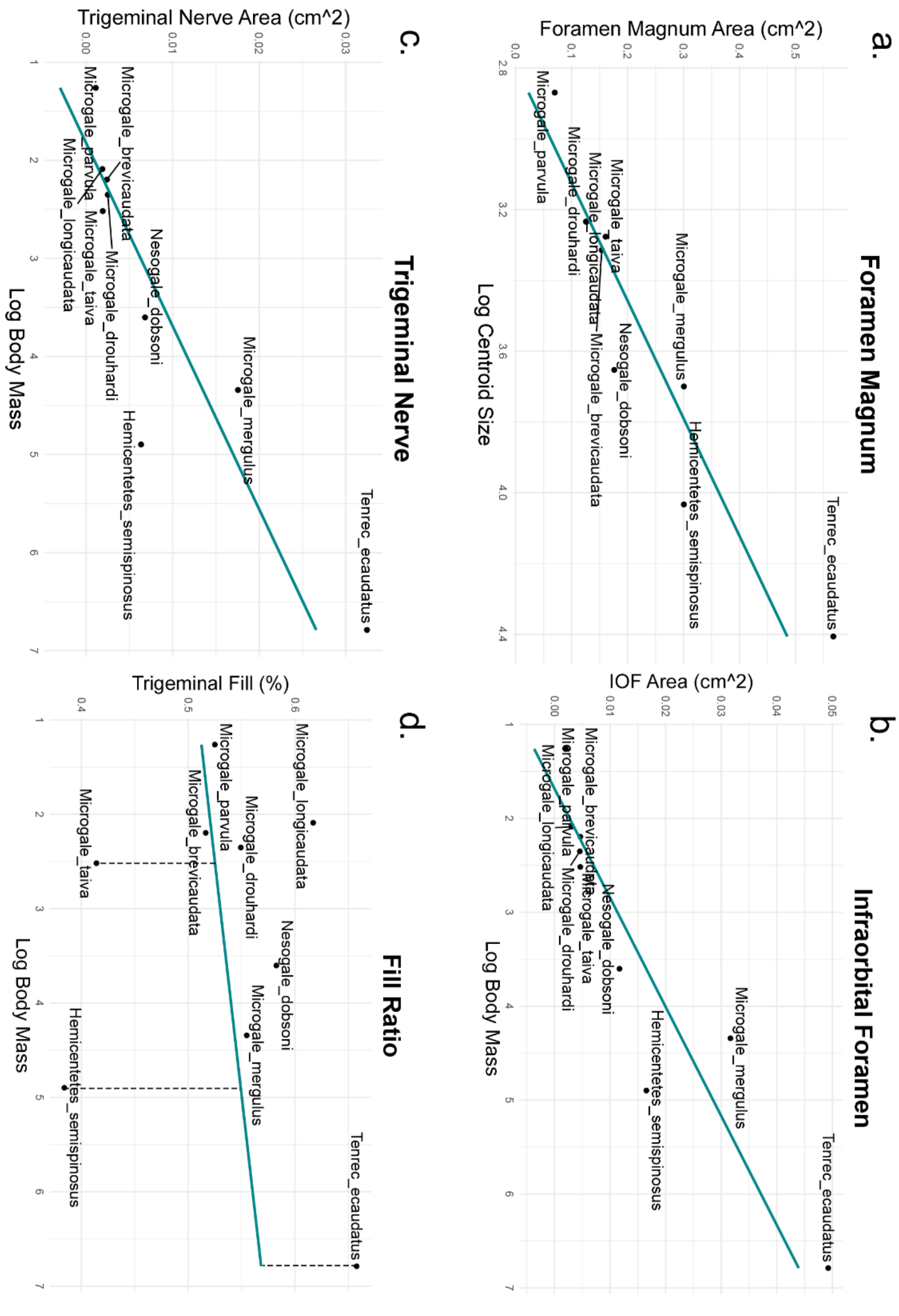

Supplementary Figure 5: Linear regressions of various bony and soft tissue indicia on log body mass. Body mass values were taken from the panTHERIA database (Jones et al 2009) and plotted against the cross-sectional area of the foramen magnum (**A**), trigeminal nerve (**B**), and infraorbital foramen (**C**). All measurements were taken utilizing the Fiducials module in 3DSlicer. A fourth regression (**D**) was run to determine correlations between the infraorbital foramen cross-sectional area and that of the trigeminal nerve. Raw data and a list of taxa sampled are available in Supplementary Data 3.

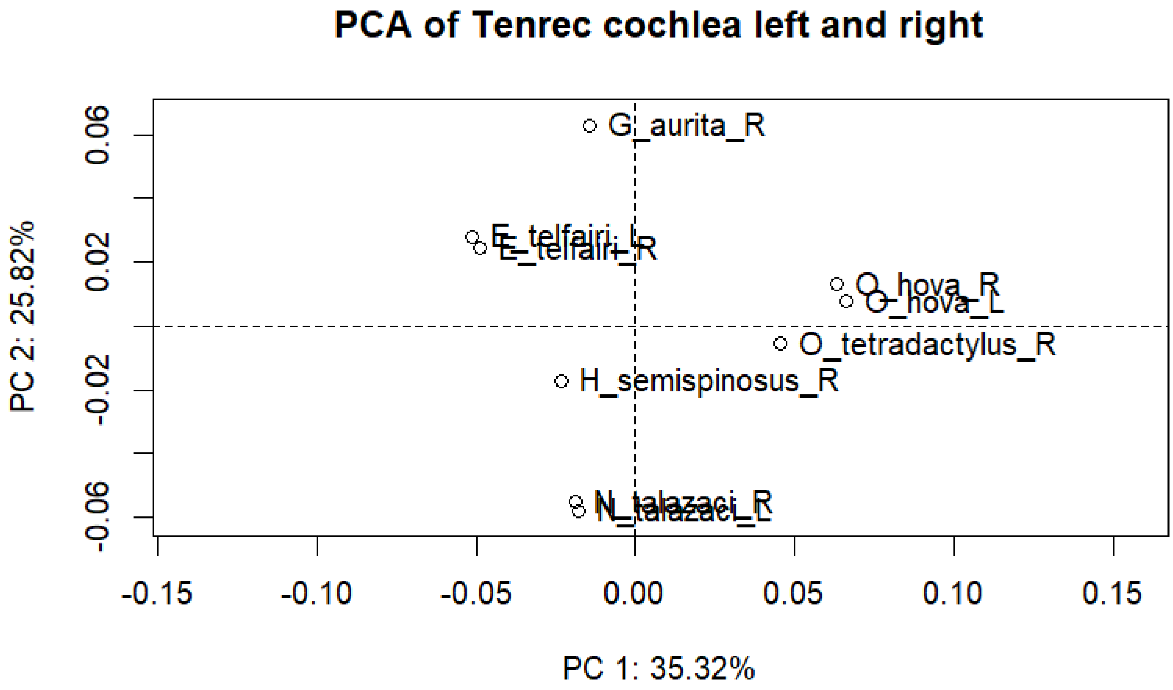

Supplementary Figure 6: Comparison of asymmetry in the tenrec bony labyrinth. Left (L) and mirrored right (R) inner ears from the same species are compared against the mirrored ears for which only the right was suitable for analyses, using the same landmarks listed in Table 2.

Supplementary Table

Table S1: Statistical results from OLS (Ordinary Least-Squares) and PGLS (Phylogenetic Generalized Least-Squares) analyses of landmarks in the tenrec inner ear. Items in **bold** correspond to statistically significant results (P values) and identified best-fit models (R squared). The final column refers to Holm adjusted P values, to account for multiple testing.

Full Ear: Single-factor ANOVA (OLS)

**Factor**	Deg. Freed.	Sum of Squares	Mean Square	R squared	F-statistic	Z-scores	P val.	P adj.
log(centroid size)	1	0.031924229	0.031924229	0.187852441	5.088673413	3.733723557	**0.001**	**0.007**
ecomorph	5	0.053777683	0.010755537	0.316445195	1.666585758	2.143079216	**0.016**	**0.032**
diet (fine detail)	6	**0.086100792**	**0.014350132**	**0.50664477**	**2.909655005**	**4.312301984**	**0.001**	**0.007**
diet (gen. categories)	2	0.040645576	0.020322788	0.239171653	3.300747624	3.314603332	**0.001**	**0.007**
locomotion	4	0.043217268	0.010804317	0.254304316	1.619890696	1.915249886	**0.028**	**0.032**
habitat	2	0.037453181	0.01872659	0.220386569	2.968213332	2.934801626	**0.001**	**0.007**
log(body mass)	1	0.03672353	0.03672353	0.216093068	6.064556021	3.891970194	**0.001**	**0.007**

Cochlea Only: Single-factor ANOVA (OLS)

**Factor**	Deg. Freed.	Sum of Squares	Mean Square	R squared	F-statistic	Z-scores	P val.	P adj.
log(centroid size)	1	0.067798	0.067798	0.147014	3.791754	2.308673	**0.011**	0.055
ecomorph	5	0.154865	0.030973	0.335812	1.82015	1.704518	**0.048**	0.144
diet (fine detail)	6	**0.232017**	**0.03867**	**0.50311**	**2.868805**	**3.079824**	**0.002**	**0.014**
diet (gen. categories)	2	0.095112	0.047556	0.206243	2.728236	2.140884	**0.019**	0.076
locomotion	4	0.114417	0.028604	0.248105	1.56737	1.221993	0.108	0.216
habitat	2	0.057086	0.028543	0.123787	1.483382	1.098366	0.145	0.216
log(body mass)	1	0.073993	0.073993	0.160449	4.204477	2.51655	**0.004**	**0.024**

Semicircular Canals Only: Single-factor ANOVA (OLS)

**Factor**	Deg. Freed.	Sum of Squares	Mean Square	R squared	F-statistic	Z-scores	P val.	P adj.
log(centroid size)	1	0.04027	0.04027	0.199886	5.496068	3.038999	**0.001**	**0.007**
ecomorph	5	0.053147	0.010629	0.263802	1.289991	1.043652	0.159	0.318
diet (fine detail)	6	**0.097426**	**0.016238**	**0.483582**	**2.653183**	**3.401462**	**0.001**	**0.007**
diet (gen. categories)	2	0.049821	0.02491	0.24729	3.449594	3.020772	**0.001**	**0.007**
locomotion	4	0.040338	0.010084	0.200221	1.189138	0.705881	0.24	0.318
habitat	2	0.048873	0.024437	0.242586	3.362957	2.876763	**0.003**	**0.009**
log(body mass)	1	0.054554	0.054554	0.270786	8.169465	3.56437	**0.001**	**0.007**

Full Ear: Single-factor ANOVA PGLS

**Factor**	Deg. Freed.	Sum of Squares	Mean Square	R squared	F-statistic	Z-scores	P val.	P adj.
log(centroid size)	1	0.001699271	0.001699271	0.06138667	1.438831844	0.872710115	0.216	0.836
ecomorph	5	0.002894565	0.000578913	0.104567014	0.420401367	-1.308582603	0.899	1
diet (fine detail)	6	0.011119912	0.001853319	0.401710048	1.902386066	1.24523716	0.111	0.555
diet (gen. categories)	2	0.006004896	0.003002448	0.216928595	2.908738884	1.590902171	0.053	0.318
locomotion	4	0.001986638	0.000496659	0.071767864	0.367254422	-1.624705898	0.947	1
habitat	2	**0.009287628**	**0.004643814**	**0.335518229**	**5.301787894**	**2.11684465**	**0.015**	0.105
log(body mass)	1	0.001921787	0.001921787	0.069425098	1.641299525	0.931501503	0.209	0.836

Cochlea Only: Single-factor ANOVA PGLS

**Factor**	Deg. Freed.	Sum of Squares	Mean Square	R squared	F-statistic	Z-scores	P val.	P adj.
log(centroid size)	1	0.001648	0.001648	0.041939	0.963038	0.361524	0.36	1
ecomorph	5	0.007151	0.00143	0.181942	0.800667	-0.0935	0.537	1
diet (fine detail)	6	**0.016102**	**0.002684**	**0.409672**	**1.966257**	**1.835427**	**0.035**	0.21
diet (gen. categories)	2	0.00633	0.003165	0.161053	2.015695	1.254383	0.112	0.56
locomotion	4	0.005275	0.001319	0.134218	0.736368	-0.24844	0.59	1
habitat	2	**0.00958**	**0.00479**	**0.243749**	**3.384278**	**1.901089**	**0.026**	0.182
log(body mass)	1	0.002702	0.002702	0.068751	1.624182	0.970226	0.177	0.708

Semicircular Canals Only: Single-factor ANOVA PGLS

**Factor**	Deg. Freed.	Sum of Squares	Mean Square	R squared	F-statistic	Z-scores	P val.	P adj.
log(centroid size)	1	0.002296	0.002296	0.078468	1.873278	0.999796	0.185	0.74
ecomorph	5	0.002864	0.000573	0.097874	0.390574	-1.71333	0.96	1
diet (fine detail)	6	0.011399	0.0019	0.3896	1.80843	1.207548	0.116	0.58
diet (gen. categories)	2	**0.006254**	**0.003127**	**0.21376**	**2.854707**	**1.656336**	**0.047**	0.282
locomotion	4	0.001918	0.000479	0.065538	0.333138	-2.11936	0.983	1
habitat	2	**0.009613**	**0.004806**	**0.328544**	**5.137656**	**2.216763**	**0.012**	0.084
log(body mass)	1	0.002016	0.002016	0.068899	1.62795	0.942903	0.2	0.74

COMPARATIVE MODELS

Full Ear: Comparative Model Test Results (OLS)

**Name**	Res. Deg. Freed.	Deg. Freed.	Res. Sum of Squares	Sum of Squares	Mean Square	R squared	F-statistic	Z-scores	P val.	P adj.
log(centroid size) = Csize	22	1	0.138019			0				
Csize + habitat	20	2	0.108722	0.029297	0.014648	0.172391	2.694639	3.023704	**0.001**	**0.007**
Csize + locomotion	17	5	0.094325	0.043694	0.008739	0.25711	1.574979	2.00122	**0.026**	0.052
Csize + ecomorph	18	4	0.103771	0.034248	0.008562	0.201527	1.485162	1.592978	0.06	0.06
Csize + diet (fine detail)	16	6	**0.071009**	**0.06701**	**0.011168**	**0.394308**	**2.516493**	**4.18393**	**0.001**	**0.007**
Csize + (gen. categories)	20	2	0.109265	0.028754	0.014377	0.169198	2.631588	2.971059	**0.002**	**0.008**
habitat	21	1	0.13249			0				
habitat + ecomorph	16	5	0.076149	0.056341	0.011268	0.331526	2.36758	3.122451	**0.003**	**0.009**
habitat + log (body mass)	20	1	0.1068	0.02569	0.02569	0.151168	4.810846	3.82347	**0.001**	**0.007**

Cochlea Only: Comparative Model Test Results (OLS)

**Name**	Res. Deg. Freed.	Deg. Freed.	Res. Sum of Squares	Sum of Squares	Mean Square	R squared	F-statistic	Z-scores	P val.	P adj.
log(centroid size) = Csize	22	1	0.393368			0				
Csize + habitat	20	2	0.361838	0.031529	0.015765	0.068369	0.871364	0.056769	0.477	0.477
Csize + locomotion	17	5	0.257747	0.13562	0.027124	0.294082	1.788997	1.459418	0.072	0.216
Csize + ecomorph	18	4	0.292501	0.100867	0.025217	0.218721	1.55179	1.101692	0.141	0.282
Csize + diet (fine detail)	16	6	**0.20085**	**0.192518**	**0.032086**	**0.417459**	**2.556045**	**2.47581**	**0.005**	**0.035**
Csize + (gen. categories)	20	2	0.315711	0.077657	0.038828	0.168393	2.459753	1.870397	0.028	0.15

habitat	21	1	0.404079			0				
habitat + ecomorph	16	5	0.236187	0.167892	0.033578	0.364061	2.274704	1.978028	**0.025**	0.15
habitat + log (body mass)	20	1	0.351547	0.052532	0.052532	0.113911	2.988606	1.899403	**0.033**	0.15

Semicircular Canals Only: Comparative Model Test Results (OLS)

**Name**	Res. Deg. Freed.	Deg. Freed.	Res. Sum of Squares	Sum of Squares	Mean Square	R squared	F-statistic	Z-scores	P val.	P adj.
log(centroid size) = Csize	22	1	0.161197			0				
Csize + habitat	20	2	0.125249	0.035948	0.017974	0.178429	2.870093	3.126867	**0.002**	**0.01**
Csize + locomotion	17	5	0.116842	0.044354	0.008871	0.220157	1.290672	1.188572	0.122	0.244
Csize + ecomorph	18	4	0.127316	0.033881	0.00847	0.16817	1.197521	0.793218	0.21	0.244
Csize + diet (fine detail)	16	6	0.090087	0.07111	0.011852	**0.352959**	**2.104911**	**3.551523**	**0.001**	**0.007**
Csize + (gen. categories)	20	2	0.135423	0.025774	0.012887	0.127932	1.903235	2.091112	**0.022**	0.066
habitat	21	1	0.152594			0				
habitat + ecomorph	16	5	0.091246	0.061348	0.01227	**0.304504**	**2.15145**	**2.82348**	**0.002**	**0.01**
habitat + log (body mass)	20	1	0.120571	0.032023	0.032023	0.158948	5.311858	4.053751	**0.001**	**0.007**

Full Ear: Comparative Model Test Results (PGLS)

**Name**	Res. Deg. Freed.	Deg. Freed.	Res. Sum of Squares	Sum of Squares	Mean Square	R squared	F-statistic	Z-scores	P val.	P adj.
log(centroid size) = Csize	22	1	0.006449			0				
Csize + habitat	20	2	0.005351	0.001098	0.000549	0.148842	2.051311	2.749216	**0.002**	**0.01**

Csize + locomotion	17	5	0.004694	0.001755	0.000351	0.237938	1.271003	1.264395	0.1	0.3
Csize + ecomorph	18	4	0.005105	0.001344	0.000336	0.182235	1.184711	0.826219	0.221	0.3
Csize + diet (fine detail)	**16**	**6**	**0.003992**	**0.002457**	**0.000409**	**0.33312**	**1.641046**	**2.802982**	**0.003**	**0.012**
Csize + (gen. categories)	20	2	0.005215	0.001235	0.000617	0.167408	2.367775	3.250587	**0.001**	**0.007**
habitat	21	1	0.13249			0				
habitat + ecomorph	16	5	**0.076149**	**0.056341**	**0.011268**	**0.331526**	**2.36758**	**3.122451**	**0.003**	**0.009**
habitat + log (body mass)	20	1	0.1068	0.02569	0.02569	0.151168	4.810846	3.82347	**0.001**	**0.007**

Cochlea Only: Comparative Model Test Results (PGLS)

**Name**	Res. Deg. Freed.	Deg. Freed.	Res. Sum of Squares	Sum of Squares	Mean Square	R squared	F-statistic	Z-scores	P val.	P adj.
log(centroid size) = Csize	22	1	0.017698			0				
Csize + habitat	20	2	0.016427	0.001271	0.000635	0.067184	0.773672	-0.15735	0.546	0.546
Csize + locomotion	17	5	0.01177	0.005928	0.001186	0.313353	1.712293	1.440288	0.089	0.415
Csize + ecomorph	18	4	0.013108	0.00459	0.001148	0.242642	1.575783	1.192163	0.128	0.415
Csize + diet (fine detail)	16	6	**0.0104**	**0.007299**	**0.001216**	**0.385817**	**1.871504**	**1.881066**	**0.029**	0.203
Csize + (gen. categories)	20	2	0.01572	0.001978	0.000989	0.10457	1.258382	0.844302	0.208	0.416
habitat	21	1	0.017578			0				
habitat + ecomorph	16	5	0.01116	0.006418	0.001284	0.339267	1.840289	1.515081	0.083	0.415
habitat + log (body mass)	20	1	0.01576	0.001818	0.001818	0.096078	2.306457	1.598935	0.06	0.36

Semicircular Canals Only: Comparative Model Test Results (PGLS)

**Name**	Res. Deg. Freed.	Deg. Freed.	Res. Sum of Squares	Sum of Squares	Mean Square	R squared	F-statistic	Z-scores	P val.	P adj.
log(centroid size) = Csize	22	1	0.008018			0				
Csize + habitat	20	2	0.006537	0.001481	0.000741	**0.165292**	**2.265685**	**3.040398**	**0.002**	**0.014**
Csize + locomotion	17	5	0.00608	0.001938	0.000388	0.216311	1.083903	0.40595	0.357	1
Csize + ecomorph	18	4	0.006539	0.001479	0.00037	0.165054	1.017762	0.109444	0.46	1
Csize + diet (fine detail)	16	6	0.00527	0.002748	0.000458	0.306647	1.390244	1.79146	**0.036**	0.144
Csize + (gen. categories)	20	2	0.006727	0.001291	0.000646	0.144111	1.919626	2.57446	**0.006**	**0.03**
habitat	21	1	0.007396			0				
habitat + ecomorph	16	5	0.005507	0.001888	0.000378	0.210757	1.097266	0.473809	0.345	1
habitat + log (body mass)	20	1	0.006645	0.000751	0.000751	0.083829	2.260866	2.666575	**0.005**	**0.03**
