# Supplementary material for: Variation and disparity within the inner ear and trigeminus of the tenrecomorpha
Source: Commun Biol. 2025 Jul 23;8:1090. doi: 10.1038/s42003-025-08489-8 (PMC12284173; doi:10.1038/s42003-025-08489-8)
Supplement: Supplementary file 2 — Description of Additional Supplementary Files [file 42003_2025_8489_MOESM2_ESM.docx]

**Description of Additional Supplementary Files**

File name- Supplementary Data 1

File description- Raw landmark and mesh data for recreating analyses.

File name- Supplementary Data 2

File description- Supplementary .CSV data for analyses.

File name- Supplementary Data 3

File description- Supplementary .CSV data for analyses.
