# Supplementary material for: Variation and disparity within the inner ear and trigeminus of the tenrecomorpha
Source: Commun Biol. 2025 Jul 23;8:1090. doi: 10.1038/s42003-025-08489-8 (PMC12284173; doi:10.1038/s42003-025-08489-8)
Supplement: Supplementary file 6 — Reporting Summary [file 42003_2025_8489_MOESM6_ESM.pdf]

nature portfolio

Corresponding author(s): Richard Benjamin Sulser

Last updated by author(s): May 16, 2025

Reporting Summary

Nature Portfolio wishes to improve the reproducibility of the work that we publish. This form provides structure for consistency and transparency in reporting. For further information on Nature Portfolio policies, see our Editorial Policies and the Editorial Policy Checklist.

Statistics

For all statistical analyses, confirm that the following items are present in the figure legend, table legend, main text, or Methods section.

n/a	Confirmed	
☐	☒	The exact sample size (*n*) for each experimental group/condition, given as a discrete number and unit of measurement
☐	☒	A statement on whether measurements were taken from distinct samples or whether the same sample was measured repeatedly
☐	☒	The statistical test(s) used AND whether they are one- or two-sided<br>*Only common tests should be described solely by name; describe more complex techniques in the Methods section.*
☐	☒	A description of all covariates tested
☐	☒	A description of any assumptions or corrections, such as tests of normality and adjustment for multiple comparisons
☐	☒	A full description of the statistical parameters including central tendency (e.g. means) or other basic estimates (e.g. regression coefficient) AND variation (e.g. standard deviation) or associated estimates of uncertainty (e.g. confidence intervals)
☐	☒	For null hypothesis testing, the test statistic (e.g. *F*, *t*, *r*) with confidence intervals, effect sizes, degrees of freedom and *P* value noted<br>*Give P values as exact values whenever suitable.*
☒	☐	For Bayesian analysis, information on the choice of priors and Markov chain Monte Carlo settings
☒	☐	For hierarchical and complex designs, identification of the appropriate level for tests and full reporting of outcomes
☒	☐	Estimates of effect sizes (e.g. Cohen's *d*, Pearson's *r*), indicating how they were calculated

Our web collection on statistics for biologists contains articles on many of the points above.

Software and code

Policy information about availability of computer code

Data collection	Skulls of 24 tenrecomorph species were scanned on the GE Phoenix Vtome x SMicroCT scanner in the AMNH Microscopy and Imaging Facility (MIF), at a target resolution between 18-56 µm. For all taxa, the left bony labyrinth of adult specimens was segmented. For 4 specimens where this structure was damaged or otherwise unsuitable for analysis, the right cochlea was segmented and mirrored prior to analysis. For rendering, specimens were reconstructed with the 3DSlicer software suite and semilandmarks placed using the SlicerMorph package. For fixed landmarks, landmarks were collected, translated from a template (Nesogale talazaci) using the ALPACA module, and corrected/ resampled by hand where necessary. Cochlear and semicircular landmarks were placed and resampled manually for each specimen, and all landmarks were merged to form the final dataset.
Data analysis	All analyses were run under R 4.4.1.

For manuscripts utilizing custom algorithms or software that are central to the research but not yet described in published literature, software must be made available to editors and reviewers. We strongly encourage code deposition in a community repository (e.g. GitHub). See the Nature Portfolio guidelines for submitting code & software for further information.

Data

Policy information about availability of data

All manuscripts must include a data availability statement. This statement should provide the following information, where applicable:

- Accession codes, unique identifiers, or web links for publicly available datasets
- A description of any restrictions on data availability
- For clinical datasets or third party data, please ensure that the statement adheres to our policy

Code and data that support the findings of this study are included in the supplementary information and available on Dryad repository (DOI: 10.5061/dryad.4qrfj6qjn). Raw image data are available on MorphoBank (to be made public pending acceptance)

Research involving human participants, their data, or biological material

Policy information about studies with human participants or human data. See also policy information about sex, gender (identity/presentation), and sexual orientation and race, ethnicity and racism.

Reporting on sex and gender	N/A
Reporting on race, ethnicity, or other socially relevant groupings	N/A
Population characteristics	N/A
Recruitment	N/A
Ethics oversight	N/A

Note that full information on the approval of the study protocol must also be provided in the manuscript.

Field-specific reporting

Please select the one below that is the best fit for your research. If you are not sure, read the appropriate sections before making your selection.

☒ Life sciences ☐ Behavioural & social sciences ☐ Ecological, evolutionary & environmental sciences

For a reference copy of the document with all sections, see nature.com/documents/nr-reporting-summary-flat.pdf

Life sciences study design

All studies must disclose on these points even when the disclosure is negative.

Sample size	N = 24 cochlear endocasts, N= 9 trigeminal nerve renders. One specimen was chosen per species.
Data exclusions	All data are included in the analyses.
Replication	All data are included to facilitate replication of the study.
Randomization	This study uses randomized residual permutation (lm.rrpp) for linear model calculations
Blinding	N/A

Reporting for specific materials, systems and methods

We require information from authors about some types of materials, experimental systems and methods used in many studies. Here, indicate whether each material, system or method listed is relevant to your study. If you are not sure if a list item applies to your research, read the appropriate section before selecting a response.

Materials & experimental systems

n/a	Involved in the study
☒	☐ Antibodies
☒	☐ Eukaryotic cell lines
☒	☐ Palaeontology and archaeology
☐	☒ Animals and other organisms
☒	☐ Clinical data
☒	☐ Dual use research of concern
☒	☐ Plants

Methods

n/a	Involved in the study
☒	☐ ChIP-seq
☒	☐ Flow cytometry
☒	☐ MRI-based neuroimaging

Animals and other research organisms

Policy information about studies involving animals; ARRIVE guidelines recommended for reporting animal research, and Sex and Gender in Research

Laboratory animals	No laboratory animals used for this study
Wild animals	Study used museum collections and no additional wild animals were used in this study. Data pertaining to original collections of these specimens are available at the corresponding institutional databases.
Reporting on sex	Study used museum collections, sex data pertaining to original collections of these specimens are available at the corresponding institutional databases.
Field-collected samples	Study used museum collections and no original field collections were used in this study. Data pertaining to original collections of these specimens are available at the corresponding institutional databases.
Ethics oversight	All specimens were loaned and scanned with permissions from their respective collections departments.

Note that full information on the approval of the study protocol must also be provided in the manuscript.

Plants

Seed stocks	N/A
Novel plant genotypes	N/A
Authentication	N/A
